# Systematic Review of Psychosocial Risk and Protective Factors in Children Reported from Developmental Criminology

**DOI:** 10.3390/children11080974

**Published:** 2024-08-13

**Authors:** Daniela Zúñiga, Francesco Carretta, Macarena Contreras, Erica Cornejo, Constanza Gallardo, Isidora Guichapani, Constansa Muñoz

**Affiliations:** 1 Institute of Psychological Studies, Faculty of Medicine, Universidad Austral de Chile, Isla Teja Campus, Valdivia 5110566, Chile; 2 Faculty of Law, Pontificia Universidad Católica de Valparaíso, Valparaíso Campus, Valparaíso 2340025, Chile; francesco.carretta@pucv.cl; 3 School of Psychology, Faculty of Medicine, Universidad Austral de Chile, Isla Teja Campus, Valdivia 5110566, Chile; macarena.contreras01@alumnos.uach.cl (M.C.); erica.cornejo@alumnos.uach.cl (E.C.); contanza.gallardo@alumnos.uach.cl (C.G.); isidora.guichapani@alumnos.uach.cl (I.G.); constansa.munoz@alumnos.uach.cl (C.M.)

**Keywords:** developmental criminology, childhood, children, protective factors, risk factors, criminality, crime, life course, systematic review

## Abstract

Background/Objectives: Evidence indicates that persistent transgressive behaviors often begin early in development and increase around age twelve, and warns that children who exhibit transgressive behaviors in childhood or early adolescence tend to develop criminal behaviors in adulthood which makes childhood a critical unit of analysis for timely intervention. The study examines risk and protective factors in childhood related to illegal behavior, through the perspective of developmental criminology. The observation of risk and protective factors in early stages allows us to design interventions that prevent social adjustment problems in children from becoming more complex by maintaining the transgression of social norms over time. Factors identified by developmental criminology can be organized according to ecological systems theory and discussed in relation to previous criminological studies. Methods: Using a systematic review based on the PRISMA method, the study identifies 24 updated developmental criminology articles that study early protective factors between birth and age twelve. Result: Risk factors at the individual level include biological, socioemotional, behavioral, symptomatic aspects and adverse life experiences. Individual protective factors include cognitive, socioemotional, and personality development aspects. Risk factors at an interpersonal and contextual level are related to family, school, peers, socioeconomic situation and governance. Conclusions: This review highlights the importance of recognizing risk and protective factors in child development, contemplating interventions at multiple levels where an articulation between the various institutions involved in child care is possible.

## 1. Introduction

Understanding criminal behavior involves examining multiple factors related to antisocial, delinquent, and criminal activities, considering their duration, frequency, intensity, and severity [1,2]. Comprehensive approaches from a specialized field enable the prevention, prediction, and detection of antisocial behavior and personality [3].

Numerous theories have contributed to explaining criminal behavior in adolescence, and it is possible to organize them into three groups: the classic theories of crime that try to explain crime from different biological, psychological, and sociological approaches; the critical or criminalization theories; and the integrative theories that are built by incorporating previous theoretical proposals, which allow us to visualize the problem of crime from a broad comprehensive framework and reflecting its complexity [4].

One theoretical integrative framework is developmental criminology, a branch built on evidence from various disciplines [5,6]; it has been considered one of the most accurate theories to understand delinquency in the juvenile stage since developmental criminology encompasses crime throughout the life course, in contrast to traditional or classic criminological theories that do not consider the passage of time, focusing particularly on childhood and adolescence [7,8,9,10,11]. From this perspective, law-breaking is viewed as part of a life trajectory that can begin early in childhood and continue into adolescence, with desistance or intensification of criminal behavior being common [12]. This approach seeks to explain the development of delinquency through an age-graded framework based on empirical observations from prospective longitudinal studies [6]. Research consistently views delinquency as part of a broader social phenomenon encompassing antisocial behavior that persists throughout the life cycle and across generations [13]. Consequently, the challenge lies in preventing and disrupting its persistence.

The literature delimits two lines of research that have in common observing life history and social development, differing in their emphases. Developmental theories, rooted in psychology, emphasize psychosocial factors, the appearance of delinquency, and the role of psychological factors. early protective and risk factors [13]. In contrast, life course theories, with a sociological orientation, focus on social structure and life events, investigating desistance and turning points [13]—for example, the research of Sampson and Laub [14] emphasizes the desistance of antisocial behavior. Both approaches, life course and developmental criminology, provide insights into various aspects of criminality that had not been previously considered and allow design preventive strategies [14].

In recent years, various reviews of the theoretical body that underpins developmental criminology have been developed [13,15,16]. McGee and Farrington [6] reviewed and compared six of the most relevant theories: the integrated cognitive antisocial potential theory [17]; the social development model [18]; antisocial behavior persistent throughout life and limited in adolescence [9,19]; the theory of informal social control by age [14,20]; the situational action theory of crime causation [21,22]; and interactional theory [23,24].

Currently, the most important theories, Farrington, Loeber, and Catalano and Hawkins, base their developments on several classic theories, such as those of social learning, social control, opportunities, differential association, and labeling, and test them through longitudinal and experimental studies [25]. Farrington, Wikström, Hawkins, and Catalano work from early socialization, postulating that children learn practices from their parents and the community during childhood [6]. Moffitt, another important theory, explores the early neuropsychological factors that result in two types of criminality: persistent and adolescent-limited [6].

In summary, current developmental criminology has managed to focus on three perspectives of analysis: (1) the development of criminal and antisocial behaviors, (2) risk and protective factors at different stages of life, and (3) the effects of certain life events in development [26]. These studies have contributed significantly to criminology, allowing preventive interventions to be designed based on evidence [25]. In relation to risk factors, it is important to point out the work of Bonta and Andrews [27], who, based on general personality and cognitive social learning theory of criminal behavior, determine psycho-social–biological factors that initiate and maintain these behaviors that are called the eight risk/need factors that are identified in meta-analysis studies; these are criminal history, procriminal attitudes, procriminal associates, antisocial personality patterns, family/marital, school/work, substance misuse, leisure/recreation. These factors are associated with the decision to engage in criminal conduct (rewards/cost favorable to crime), mediated by four factors (called the big four) that come from family, neighborhood, gender, age, and ethnicity [27].

Within developmental criminology, there has been a priority in the study of risk and protective factors in adolescents, possibly due to the minimum age of juvenile criminal responsibility that, in most countries, coincides with this stage of the life cycle. Childhood is receiving less attention in research, which, from a life course perspective, spans from birth to 12 years of age [28]; however, evidence indicates that persistent transgressive behaviors often begin early and increase at age 12. Farrington [29] warns that children exhibiting transgressive behaviors in childhood or adolescence tend to develop criminal behaviors in adulthood, making childhood a critical unit of analysis for timely intervention.

Children are not criminally responsible until the minimum age of criminal responsibility established in each country, and they require different treatment than adults focused on protection [30], but few studies describe the factors involved when illicit behavior begins at early ages (between four and seven years), the period in which these behaviors tend to begin [26]. Identifying early biopsychosocial mechanisms could help prevent children from starting a trajectory of chronic antisocial behavior that continues into adulthood [31].

Therefore, it is crucial to understand the risk and protective factors underlying children’s social behaviors, as these could predict and antisocial or prosocial behavior. These factors include individual characteristics and the social environment in which children develop, with the family being the primary reference, along with friends, school, and society [25,32].

Risk factors include a conflicting and disorganized bond between the child and caregivers, discipline techniques applied by caregivers, having friends involved in delinquency, and certain individual psychological and moral factors acquired and developed in childhood that may relate to social adaptation [25,33]. However, despite exposure to the same risk factors, some children do not develop criminal behavior due to individual and environmental variables that function as protective factors against severe cumulative events and stressful situations. These protective factors refer to individual characteristics and the family or community context that can mitigate the effect of risk factors, increasing resilience and preventing the development or persistence of such behaviors [25]. One such factor is guilt related to morality, which reinforces self-control in high-criminal temptation scenarios [33].

Taking the above into account, focusing on children is crucial due to their greater capacity for change and flexibility. Most individuals with persistent criminal behavior began their activities at an early age, closely related to developmental contexts [25]. Observing risk and protective factors allows us to design interventions that prevent social adjustment problems in children from becoming more complex by maintaining the transgression of social norms [9,34].

The factors identified by developmental criminology can be organized according to the theory of ecological systems [35], which proposes that human behavior is understood in a dynamic relationship with other systems, distinguishing from the close ones (microsystem) to the most distant ones (macrosystem, exosystem) [36]; classifying them from this point of view could contribute to understanding the criminal behavior of children and adolescents considering the contexts, situation, and social position. In addition, it is possible to consider individual resources, community, and social needs and the articulations between systems. Behavioral changes require a collaborative effort between different systems of which children, adolescents, and adults responsible for care and interventions will have less or more control, so it is possible to assess which elements are under the one domain of children and those who accompany their development (family, school, health, and justice systems, among other systems), as well as factors that cannot be addressed immediately and that require long-term structural change. Classifying the risk and protective factors described in the updated literature from the perspective of developmental criminology and considering the person and transactions with the context (Person-Process-Context-Time Model of Development) or what Sheldon calls “Develecology,” such as “the study of the processes of development of organisms and their changing relations with their environments, employing a combination of systemic and longitudinal perspectives that include the mutual and reciprocal transactions of organism and context” [37], could lead to reflection about when to intervene, the social systems involved, and how they are articulated. If the models proposed from criminology dialogue with the current systemic health and educational models, it would be possible to advance in multisystemic, comprehensive, and collaborative interventions between different agents, since the justice system by itself will not be able to solve the social problem of crime in childhood and youth [35].

Given the complexity of addressing crime in childhood, the objective of this study arises: to analyze the existing updated empirical studies on psychosocial factors in developmental, risk, and protection criminology in relation to crime in childhood and then classify them according to the ecological model. The specific objectives are: 1. Describe the articles found by country, year of publication, and methodology; 2. Identify the protective and risk factors in the analyzed texts; 3. Categorize protective and risk factors at systemic levels: individual, interpersonal, and contextual; 4. Identify early behavioral factors, from birth to 7 years, reporting on updated literature.

## 2. Materials and Methods

This research is a systematic review, which involves summarizing evidence by one or more experts on a specific topic, identifying, evaluating, and synthesizing it to answer a particular question and draw conclusions from the collected data [38]. In this study, an analysis is conducted on psychosocial factors related to criminality in children, selected under predefined inclusion and exclusion criteria.

To ensure the validity and quality of the systematic review, the PRISMA methodology was used (Preferred Reporting Items for Systematic Reviews and Meta-Analyses). Yepes-Núñez et al. [39] in “PRISMA 2020 Statement: An Updated Guideline for Reporting Systematic Reviews” indicated that the PRISMA Statement has been developed for systematic reviews to assess the effects of health interventions regardless of the design of the selected studies. It is noteworthy that PRISMA 2020 can be applied to original, updated, or continuously updated systematic reviews. 

### 2.1. Study Selection

A bibliographic search was conducted during October and November 2023, selecting empirical quantitative and qualitative scientific articles from indexed journals that indicate psychosocial factors in children, as reported in the literature from 2017 to 2023. For the systematic review, the following elements were included:

(“Developmental criminology” OR “life course criminology”) AND (“risk factors” OR “protect factor” OR “protective factors”).

And for EBSCOhost:

Developmental criminology OR life course criminology AND protective factors OR risk factors.

### 2.2. Inclusion and Exclusion Criteria 

This systematic review includes articles published in popular science journals. Articles from the following databases are considered: SCIELO, SCOPUS, Web of Science, PubMed, and EBSCOhost, from those open access repositories in English, Portuguese, and Spanish. Articles were included whose study subjects belonged to the age group of 1 to 12 years. Empirical methodology articles related to the topic and published between 2017 and 2023 were included due to the need for updated information on psychosocial factors. The exclusion criteria exclude articles not related to the topic, articles that are not open access in languages other than English, Portuguese, and Spanish, as well as books, press articles, essays, theses, reports, opinion columns, and conference papers. The filters applied in the search databases are empirical studies, the year range from 2017 to 2023, language as indicated in the inclusion criteria, and open access articles. The selection and data collection process conforms to the PRISMA model and is organized in the following steps: 1. A search for articles is carried out in SCIELO, SCOPUS, Web of Science, PubMed, and EBSCOhost. 2. One researcher reviewed the articles from each database, totaling five researchers, one for each database. 3. All researchers use the same ad hoc instrument, an Excel database, where each sheet corresponds to an inclusion criterion. In a systematic and sequential manner, it is possible to reach the results. This standardized procedure helps reduce bias in the results and ensures reliability.

## 3. Results

**Figure 1:**
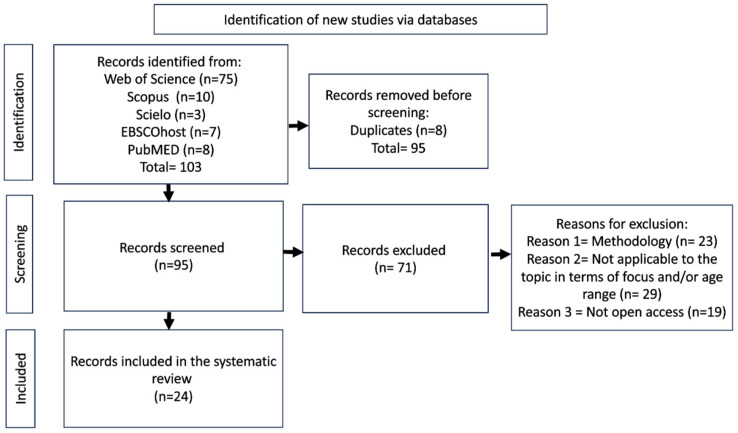
Search identification flow diagram. Based on the PRISMA Statement.

The systematic review results can be seen in Figure 1. After applying the exclusion criteria, 24 articles were reviewed, allowing us to respond to our objectives.

### 3.1. Article Characterization

Regarding the methodology, 23 of the selected articles have a quantitative approach, and one article employs a mixed-method approach. Concerning the design, 22 articles are longitudinal, and two are cross-sectional. Among the analyzed articles, 50% use a database of male participants, 42% are mixed, and 8% are female only.

The geographical distribution of the reviewed studies shows that 13 are from North America, with 11 from the USA and 2 from Canada. Only one study is from Latin America (Brazil).

In terms of publication years, the distribution is as follows: seven articles from 2021; five from 2020; four from 2019; four from 2017; and one each from 2018, 2022, and 2023. 

### 3.2. Contents of the Selected Articles 

**Figure 2:**
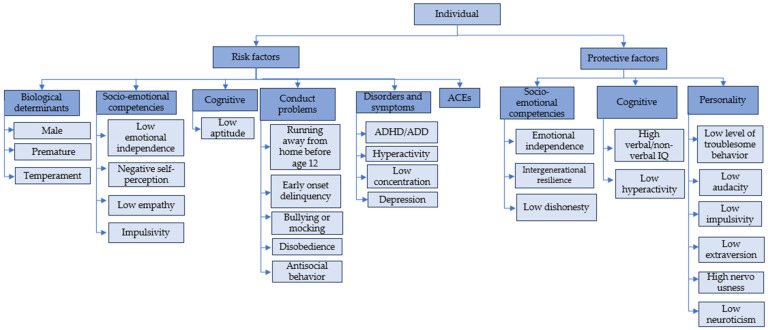
Individual-level content.

**Figure 3:**
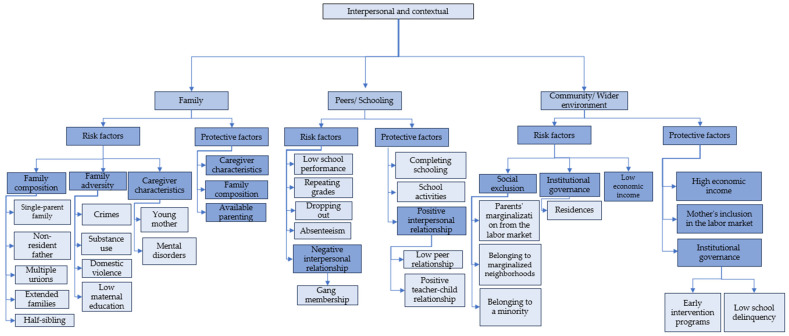
Interpersonal and contextual-level content.

To address the proposed objectives, the 24 selected articles are organized into content trees for each systemic level of analysis: individual, interpersonal, and contextual. Bronfenbrenner’s ecological theory is used as a basis for categorization, with the individual level being everything related to the child’s characteristics (Figure 2), while the interpersonal and contextual (Figure 3) encompass the microsystem and mesosystem (family, peers/school), in addition to the exosystem and macrosystem (community/wider environment). Each tree includes protective and risk factors in childhood with respect to delinquency according to updated literature on developmental criminology.

#### 3.2.1. Individual Risk Factors 

The individual risk factors reported in the literature include biological determinants, socio-emotional competencies, cognitive factors, conduct problems, psychopathological disorders and symptoms, and adverse childhood experiences (see Figure 2).

In the category of biological determinants, Van Hazebroek et al. [40] mention that being male is associated with antisocial behaviors and higher recidivism in serious violent and chronic (SVC) crimes [41]. Herrera and Stuewig [42] also note that being male is a risk factor, especially when interrelated with other components such as witnessing spousal violence (for adolescent delinquency) or living in violent contexts with substance use. Regarding preterm birth, Givens and Reid [43] link it to early-onset delinquency. Additionally, temperament, specifically “hyperreactivity” and a “short temper,” increases the likelihood of SVC infractions in adulthood [41]. Neuroticism is also identified as a risk factor for the development of chronic persistent delinquency, characterized by younger age at first conviction and longer criminal careers [44].

The socio-emotional competencies category highlights low emotional independence from an incarcerated father and negative self-perception as risk factors. The former facilitates intergenerational continuities in crime [41], while the latter increases the likelihood of developing RORD and slow-rising chronic (SRC) delinquent trajectories [45]. Impulsivity, referring to low self-control, can lead to antisocial behaviors [46,47]. Low empathy is linked to sexual crimes in adolescence [48].

Cognitive factors include low aptitude, referring to low verbal and non-verbal IQ, which is an early risk characteristic for delinquency [44,46,49].

Conduct problems refer to early behaviors that are indicators of the possible development of the disorder in its entirety [50]. Gushue et al. [45] mention that running away from home before age 12 increases the likelihood of quickly engaging in and desisting from delinquency (Rapid Onset Rapid Desistance, RORD).

Early-onset delinquency is another critical factor. Authors such as Han and Park [51] and Wolff [41] indicate that delinquent behaviors in childhood increase the risk of lifelong delinquency. Solomon et al. [52] identify bullying, mocking others, and disobedience as factors that can lead to antisocial behaviors in adulthood. Koegl et al. [53] and Solomon et al. [52] also mention that such behaviors in children are related to delinquency in adolescence and adulthood.

The category of disorders and symptoms refers to psychopathological indicators. Rosa et al. [48] mention that attention deficit hyperactivity disorder (ADHD) is linked to nonsexual crimes. Solomon et al. [52] and Whitten et al. [44] indicate that low concentration levels and hyperactivity are predictors of persistent delinquency, understood as those with older age at first conviction but longer criminal careers. Depression also influences serious crimes in both women and men [42].

Finally, adverse childhood experiences (ACEs) can act as risk factors according to Craig et al. [54]. The study indicates that the more ACEs a child has, the more likely they are to commit crimes in late adulthood. However, the number of ACEs was not predictive in women with SVC crimes [41].

#### 3.2.2. Individual Protective Factors 

The factors are divided into socio-emotional competencies. Socio-emotional competencies include high emotional independence from incarcerated parents [41], low dishonesty [53], and intergenerational resilience in children. Intergenerational resilience refers to a reduction in crime across generations within the same family, mediated by various protective elements that manifest in each age group. However, since it is a recent concept, it lacks detailed factors mediating its appearance [55].

Concerning cognitive factors, Craig et al. [54] and Capaldi et al. [56] indicate that high verbal IQ (vocabulary and reading) significantly reduces the likelihood of delinquency in males. This factor, along with low hyperactivity, also protects against ACEs [56].

Finally, in terms of personality, Craig et al. [54] identify low troublesome behavior, described as “being unproblematic”, as a protective factor against delinquency. Additional elements that significantly reduce the risk of committing a crime include low audacity, low impulsivity, low extraversion, high nervousness, and low neuroticism [54].

#### 3.2.3. Family Risk Factors

Family composition factors identified as risk factors include single-parent families, non-resident fathers, having half-siblings, and multiple maternal unions before childbirth, with the most prevalent factor being a non-resident father [52]. Criminality increases with the number of these components in the family [52]. Basto-Pereira and Farrington [49] identify that extended family, along with other factors, can predict the criminal behaviors of the “serious and persistent versatile” group, characterized mainly by committing violent and, to a lesser extent, minor crimes. Finally, Whitten et al. [44] indicate that this risk element, along with high audacity, predicts chronic criminal behaviors.

The authors report a set of situations related to family adversity (see Figure 3). Having a convicted father influences the appearance of chronic persistent delinquency [44]. Similarly, Han and Park [51] indicate that substance use by caregivers is a risk component related to early alcohol and drug use in children.

Family violence and spousal violence are significant factors. Herrera and Stuewig [42] indicate that both are related to delinquency in adolescence, as minors are socialized to imitate behaviors that transgress social limits. Low maternal education (less than nine years of completed schooling) [52], child maltreatment as corporal punishment [57,58], severe or authoritarian parenting, and discipline [58] are predictors of delinquency from childhood to adolescence. Farrington [58] also mentions that caregiver neglect, understood as low parental involvement in their children’s education, is an indicator for persistent delinquency. Similarly, Whitten et al. [44] indicate that low supervision over children’s whereabouts and actions predicts longer criminal careers. Martins et al. [59] report that low stimulation in children under four correlates with the manifestation of violent crimes.

Regarding sexual abuse, Rosa et al. [48] link childhood sexual abuse to the early onset of sexual delinquency.

Regarding caregiver characteristics, Solomon et al. [52] and Basto-Pereira and Farrington [46] identify young mothers as a risk component, predicting minor crimes. Martins et al. [59] indicate that mothers with mental disorders influence the appearance of violent crimes.

#### 3.2.4. Family Protective Factors 

Family protective factors include caregiver characteristics, such as the older age of the mother; family composition, which refers to reduced family sizes; and available parenting, which points to high parental interest in education. Craig et al. [54] indicate that these elements are associated with a lower prevalence of committing crimes between the ages of 10 and 56. Reduced family sizes and/or older parents significantly reduce criminal behaviors [54].

#### 3.2.5. Peers/Schooling Risk Factors

Regarding schooling, studies such as Koegl et al. [53] indicate that low academic performance is related to future antisocial behavior. Similarly, Basto-Pereira and Farrington [49] identify low academic achievement as a predictor. Whitten et al. [44] also highlight low performance and interrupted schooling trajectories, which are linked to chronic delinquency characterized by shorter criminal careers but with a higher number of crimes and convictions. Additionally, youths who repeated grades three times and/or dropped out of school were twice as likely to commit violent crimes [59]. School absenteeism in women also constitutes a risk factor [45].

Educational environments with high crime rates and the development of links with delinquent peers are mentioned [51], representing a short-term risk for criminal behavior.

Peer risk factors include negative interpersonal relationships—for example, gang membership is linked to delinquent trajectories in incarcerated youth [60] and women in juvenile residences with SVC delinquency [41].

#### 3.2.6. Peers/Schooling Protective Factors

Regarding schooling, completing education, even with grade repetition, serves as a protective element by preventing the development of violent and nonviolent criminal careers [59], predominantly in males [54]. School activities are also protective against substance use in children [51].

Peer protective factors include positive interpersonal relationships, such as low peer relationships [54]. A positive relationship between teacher and child is also significant, allowing for the development of positive affection and the construction of healthy interactions with peers [57].

#### 3.2.7. Community Risk Factors 

Community risk factors include social exclusion, comprising three aspects (see Figure 3): exclusion from the labor market for both parents [52], belonging to marginalized neighborhoods, and minorities that may be racial, ethnic, or gender-based [40].

Regarding institutional governance, authors such as Yang et al. [60] and Valdivia-Devia et al. [61] indicate that attending residences reduces the possibility of experiencing positive turning points. Finally, low economic income, defined as situations where none of the child’s parents or caregivers receive a work income [52].

#### 3.2.8. Community Protective Factors 

Community protective factors including high economic income, which includes the mother’s inclusion in the labor market, is another protective factor [54].

Children exposed to higher risk factors who do not engage in delinquency tend to have few friends. In terms of governance and institutionalization, early multicomponent intervention programs [18] promote prosocial behaviors and prevent problematic behaviors. Finally, Craig et al. [54] identify a low school crime rate as a protective factor. 

The last specific objective raises the need to identify factors before the age of 7. Few studies have been found, with the following being noted: antisocial behaviors, physical aggression, noncompliance with rules, low child responsiveness, high hyperactivity, and impulsiveness.

## 4. Discussion

This study aimed to analyze existing empirical studies on psychosocial factors in the field of developmental criminology, such as risk and protective factors related to crime in children, incorporating an ecological perspective. As a result of the review, 24 updated articles were identified. The factors in each of the reviewed texts are categorized into different levels: individual, interpersonal, and contextual. These are visualized in Figure 2 and Figure 3.

The review shows that crime in childhood is a complex phenomenon, influenced by several factors throughout the life cycle [13]. Developmental criminology studies highlight this complexity by providing a longitudinal view of various areas of child development, allowing individual, relational, and contextual aspects to be considered to understand criminal behavior. From this perspective, children who exhibit delinquent behavior in childhood or adolescence are more likely to engage in delinquency in adulthood [29], making childhood an opportune time to intervene.

Risk factors usually act early, and some disruptive behaviors manifest before the age of seven, such as antisocial behavior, physical aggression, noncompliance with rules, low child responsiveness, high hyperactivity, and impulsivity [43,44,53,61]. Various studies highlight childhood and its care context as critical points for the possible development of criminal behavior in adolescence and/or adulthood [62,63,64,65,66,67].

Proposing interventions involves establishing conducive care and attention environments for children that promptly and adequately address behavioral problems in childhood. However, the child’s context is socially conditioned by a culture that does not emphasize care [68]. Given that young people who exhibit criminal behavior often come from vulnerable contexts and experience childhood victimization, it is understood that the cycle of violence does not occur in a vacuum [69,70,71,72].

A systematic analysis of the updated literature reveals a dynamic link between several factors identified in the development of criminal behavior. At the community level, risk factors such as labor market exclusion and low income indicate material deprivation and vulnerability [52]. This is connected to the family level, where parental crime [44] and family violence are primary elements within a context of vulnerability. However, this vulnerability is not static, reflecting the possibility of intervention and prevention through economic, labor, and social opportunities that promote contexts of early development and inclusion for children and their families.

Schooling constitutes both a protective and risk factor at individual and community levels. Situations of schooling at the individual level, such as repeating grades [59] and high school crime rates at the community level, relate to developing delinquent behaviors in children [51]. However, schooling can be a significant protective factor when individuals participate in school activities [57] and when there is a low school crime rate [54], as well as when children form meaningful bonds with their teachers. This reflects the importance of strengthening the individual educational experience from an early age while also promoting communication and inclusion of families in the school community.

From this perspective, protective and risk factors at different levels are dynamic conditions in the life history that can become more complex or resolved. To prevent early antisocial behaviors, it is essential to observe the available evidence, generate research from an evolutionary and systemic approach, and consider the experiences, learnings, and contexts in which children develop. It is also crucial for justice actors to prevent actions reported in the literature that could result in victimization and risk factors, such as institutionalization as a protection measure. Its application should be exceptional and not preferred, as living in residences or foster homes constitutes a significant risk factor for chronic crimes despite exposure to other risk factors [60].

The literature indicates that an important protective factor is working from early childhood to promote prosocial actions through activities that include the entire family unit. Parenting styles significantly influence the socio-emotional and moral development of children and, consequently, the behaviors they will exhibit throughout their lives [33,51]. Intervention and prevention strategies should focus on reducing risk and promoting factors that promote resilience. We believe that an appropriate strategy that is consistent with developmental criminology and evidence is a multisystemic intervention since factors are observed at different levels related to each other and change over time [47].

Although preventive efforts focus on adolescence, they could be more effective if it is recognized that the complexity of crime has its roots in childhood [73] and that it is crucial to address children’s adverse experiences in their early stages, as they are related to criminal behavior at later stages of development [74]. This is consistent with Bonta and Andrews [27], who emphasize understanding criminal behavior as the result of a series of factors that require attention and that influence the cost–benefit evaluation that people make when starting and maintaining crime. In the case of childhood, decision-making has its limitations due to the characteristics of children’s development and their life contexts, which possibly has a greater influence in the early stages of development due to its relationship with learning. Unlike the eight risk/need factors, this study, by focusing on childhood, allows us to observe other recent elements of importance for children: the presence of adverse childhood experiences, the early appearance of behavioral problems in close environments, and cognitive and affective difficulties. Substance consumption is not yet relevant at this stage; even so, it is logical to think that as children begin and maintain transgressive behaviors, substance consumption can begin constituting a risk factor.

According to the characterization of the articles, there is a lack of research on this approach in Latin America [59]. It is necessary to investigate the nuances for Latin America, considering that studies carried out on adolescents in Brazil [59] and Chile establish a more active role of family risk factors than in Anglo-Saxon studies [75,76,77]. Furthermore, the samples focus mainly on men, although social factors may act differently in girls and boys; this requires further studies [41,42].

Focusing on childhood when studying crime from a complex or integrative and multilevel perspective is essential. Addressing it requires evidence-based actions and a collective commitment from various institutions and social actors to build a society that cares for and respects the rights of children and their families. This study makes it clear that family, school, justice, and health systems should work collaboratively with children and their families when some of the individual, relational, and contextual factors occur, implementing strategies with families to support them socially, if required, so that it is possible to exercise their care functions, without neglecting structural aspects such as poverty, prejudice, stigmatization of minority social groups, neighborhood conditions, and the relationship that families and children build with justice actors.

The results allow us to reflect on the relevance of legislative proposals that aim to reduce the age of criminal responsibility of children in an attempt to prevent criminal behavior; evidence suggests that increasing the severity of penalties is not an effective method to prevent crimes, and if the crime rate committed by adolescents increases, the severity of sanctions will also increase, causing a downward spiral phenomenon. Studies have shown that incentives can work better than punitive measures. Therefore, timely detection of risk factors that facilitate children’s criminal behavior is essential for effective prevention [41]. Solutions to juvenile delinquency should focus on promoting research to deepen the understanding of this phenomenon and implementing preventive mechanisms based on such research findings, rather than toughening sanctions.

This systematic review covers studies from a limited time period, reviewing only articles from 2017 to 2023, consideredupdated evidence in the population of children involved in criminal behavior. However, this may leave out relevant evidence prior to 2017. Additionally, certain languages are included in this review, excluding studies that do not meet the inclusion criteria. This is a limitation of our study because research in other languages and other age groups, older than 12 years, may be left out of this review. Future systematic review studies could address gender differences, articles from previous years, and incorporating other languages and theoretical perspectives.

## Data Availability

All data are included in the article or in the tables.
